# Miscellaneous

**Published:** 1888-02

**Authors:** 


### MISCELLANEOUS.

TWENTY-FIVE years Experience with the most perfect success, has proved Winchester's Hypophophite of Lime and Soda to be a certain cure for Consumption. Coughs, Colds, and diseases of the Throat and Lungs. It promptly arrests the progress of the disease, relieves the cough, checks the exhausting night sweats, stimulates the appetite, increases the vital strength and nervous energy and causes all the general symptoms to rapidly disappear. Can be procured at all Druggists.

Persons troubled with a tendency to stoop and who are becoming round shouldered, are advised to walk with the palms of the hands forward, the thumbs outward. It will do wonders toward straightening a bent form, as any soldier will testify.

$371.21 FOR A GUESS.—The readers of our paper will be interested in knowing that the proprietors of "Warner's Log Cabin Remedies" will pay $371.21 in cash for the best answer to the question: "What is the hole for, that is in the outside of the chimney of the old fashioned log cabin, as represented in the trade-mark of Warner's Log Cabin Remedies?" A pamphlet with a picture of such a log cabin can be procured at any drug store. The answers must be sent by mail to H. H. Warner & Co., proprietors of the celebrated "WARNER'S SAFE CURE," Rochester, N. Y., before April 10th, 1888. But *one answer* from each contestant will be considered. It must be *signed with the real name*, giving post-office address, and must state that the party *has purchased and used* at least one of the following remedies: Warner's Log Cabin Sarsaparilla, Warner's Log Cabin Hops and Buchu Remedy. Warner's Log Cabin Cough and Consumption Remedy, Warner's Log Cabin Extract, Warner'sLog Cabin Liver Pills, Warner's Log Cabin Rose Cream (for catarrh, etc.). Warner's Log Cabin Scalpine (for the scalp and hair), Warner's Log Cabin Plasters. The answers will be referred to an impartial committee for decision, which will be announced April 10th, 1888. Letters of inquiry will not be answered.

Dr. I. N. LOVE, Consulting Physician to City Hospital and Editor of the *Weekly Medical Review*, of St. Louis, in a paper published in the *Review*, December 31st on "Diet in Intestinal Diseases of Children," thus alludes to the benefit to be derived from the use of BUSH'S FLUID FOOD or BOVININE, he having tested it in his own family.

"As an aid to the nutrition of the child, whatever be the form of food given, I have found great satisfaction in the administration of a raw liquid meat food known as Bovinine. In the most delicate conditions of the alimentary canal, in all stages of innutrition from the most light down to even typical cases of marasmus, I have given the Bovinine in doses ranging from five drops to a teaspoonful diluted with five or six times the amount of water every two to four hours, with marked benefit. We often have to discontinue all milk food, and in such cases I have given the Bovinine for weeks at a time exclusively.

I consider it an invaluable aid in these infantile cases, as well as in all forms of wasting disease of adult life. I base my conclusions upon practical observation in a large number of patients and favorable experience in my own family.

A crown will not cure the headache, nor a golden slipper the gout, but if the Headache comes from Catarrh, Warner's Log Cabin Rose Cream will give immediate and lasting relief. It is the best remedy.

THE NATURAL GAS.—Many theories of the formation of natural gas have often been proposed ; but it is none the less-interesting to quote here that suggested by Professor Wurtz nearly seventeen years ago in these words : "As to my views of the mode of the formation of gas that exists now in such enormous compression in the different strata, I ask first, What is this gas chemically ? Always essentially, from whatever horizon obtained, it is marsh gas, that hydrocarbon of all others which contains the most hydrogen and the least carbon ; the compound which naturally and necessarily forms the final residue of the abstraction of carbon from organic matter by a powerful oxidizing agent, since in Nature we scarce find elementary hydrogen as such a residue. Now, what oxidizing agents are there, or rather, what have there been in all these rocks that could effect such a combustion ? I reply, oxides of iron, now represented in these rocks by iron sulphides, showing the iron oxides to have passed through the forms of sulphates ;" an action similar to that "evolution of marsh gas going on in every stagnant pool, loaded with vegetable matter, and blackened by sulphide of iron, which is occupied in conveying the oxygen of the water to the carbon of the mud."

The development of the natural gas industry during the past two years has been marvelous ; yet it is almost as extraordinary that it required fifteen years after Professor Wurtz's prediction to awaken even enterprising men to what they all now know to be so incalculably important.—*Engineering and Mining Journal.*

I PITY from my heart the unhappy man who has a bad wife. She is Shackles on his feet, a Palsy to his hands, a burden on his shoulders, Smoke to his eyes, Vinegar to his teeth, a Thorn to his side, and a Dagger to his heart.—*Osborne.*

### THE LUCKY-BONE.

Oh, what shall my wish be—what shall it be ?
The lucky-bone's dry on the crane,
And Clara, sweet Clara, is bantering me
To break it, with, her in twain :
In spite of my art
She'll get the long part,
And laugh at me when I complain.

I wish Clara'd wish as *I'm* wishing to-day,
Nor mind how the lucky-bone breaks,
And ever and on by our pulling one way,
Avoid all our single mistakes,
But the sly little elf
Keeps all to herself,
The wishes she secretly makes.

